# Bisegmentectomy 7–8 for Small-for-Size Remanant Liver for Cirrhotic Patients Under Right Hemi-hepatectomy With Hepatocellular Carcinoma: A Case-Matched Comparative Study

**DOI:** 10.3389/fsurg.2021.675666

**Published:** 2021-07-15

**Authors:** Xishu Wang, Yongrong Lei, Hongbo Huan, Shu Chen, Kuansheng Ma, Kai Feng, Wan Yee Lau, Feng Xia

**Affiliations:** ^1^Institute of Hepatobiliary Surgery, Southwest Hospital, Army Military Medical University, Chongqing, China; ^2^Faculty of Medicine, Prince of Wales Hospital, The Chinese University of Hong Kong, Sha Tin, China

**Keywords:** hepatocellular carcinoma, liver cirrhotic patients, right hemi-hepatectomy, bisegmentectomy 7–8, survival

## Abstract

**Aim:** To compare the short- and long-term treatment outcomes of bisegmentectomy 7–8 vs. right hepatectomy for patients with hepatocellular carcinoma and cirrhosis.

**Methods:** Thirty six cirrhotic HCC patients with infiltration of right hepatic vein in segments 7–8 underwent bisegmentectomy 7–8 for small-for-size remanant liver under right hemi-hepatectomy. Its outcome was compared with a case-matched control group of cirrhotic HCC patients who underwent right hemi-hepatectomy during the study period.

**Results:** The study group consisted of 36 patients and the control group 36 patients selected from 1,526 patients matched with age, tumor size, tumor location, and Pugh-Child staging. There were no significant differences between the two groups in operative parameters and in perioperative main complications which included hemorrhage, bile leakage, ascites, pleural effusion, and liver failure. The overall morbidity rate and morbidity rate classified according to Clavien's classification were similar. There was no in-hospital mortality or 90 day post-operative mortality. The mean follow-up was 30 and 32 months for the study group and control group, respectively. The disease free survival rate (DFS) for the study group was just significantly better than the control group. The median DFS was 24 months for the study group and 8 months for the control group (*P* = 0.049). Meanwhile, the median cumulative overall survival was 35 months for the study group and 27 months for the control group (*P* = 0.494).

**Conclusion:** Bisegmentectomy 7–8 was safe and feasible for selected cirrhosis patients, and did not increase the perioperative risk and inferior long-term overall survival outcomes. It extended the indications for liver resection in patients with borderline volumes of future liver remnant for HCC cirrhotic liver.

## Introduction

Hepatocellular carcinoma (HCC) represents one of the most common cancers with rising incidences worldwide (1–3). Liver resection has emerged as the primary treatment in carefully selected HCC patients with cirrhosis, especially as a consequence of the current hepatic graft shortage globally (2, 4–7). With advances in surgical and radiological techniques, outcomes of liver resection in patients with cirrhosis have improved remarkably (7, 8). Preservation of non-tumorous liver parenchyma is a priority for cirrhotic patients to reduce the risk of life-threatening postoperative liver failure in cirrhotic patients, partial liver resection should be performed when the volume of the future remnant liver (FLR) is more than 50% of the total functional liver volume on hepatic volumetric studies (9–12). Bisegmentectomy 7–8 is an unusual but safe procedure which allows curative resection without unnecessary sacrifice of functional hepatic parenchyma for colorectal liver metastases. The presence of a malignant lesion in segments 7–8 with infiltration to right hepatic vein is usually considered as an indication for right hemi-hepatectomy (13). Nevertheless, when volumetric studies suggest the volume of the FLR to be inadequate to support right hemi-heptectomy, especially in patients with cirrhosis, bisegmentectomy 7–8 can still be performed in patients with a prominent inferior right hepatic vein shown on angiography or contrast enhanced CT. However, anatomic studies showed that a prominent inferior right hepatic vein (RHV) draining segment 6 is present in ~1/4 to 1/2 of patients (14). As the efficacy and safety of bisegmentectomy 7–8 for cirrhotic patients with HCC have only been reported in few studies with short-term survival outcomes, the present study aimed to evaluate the feasibility, efficacy, and safety of bisegmentectomy 7–8 in the treatment of these patients.

## Patients And Methods

### Patients

**Figure 1:**
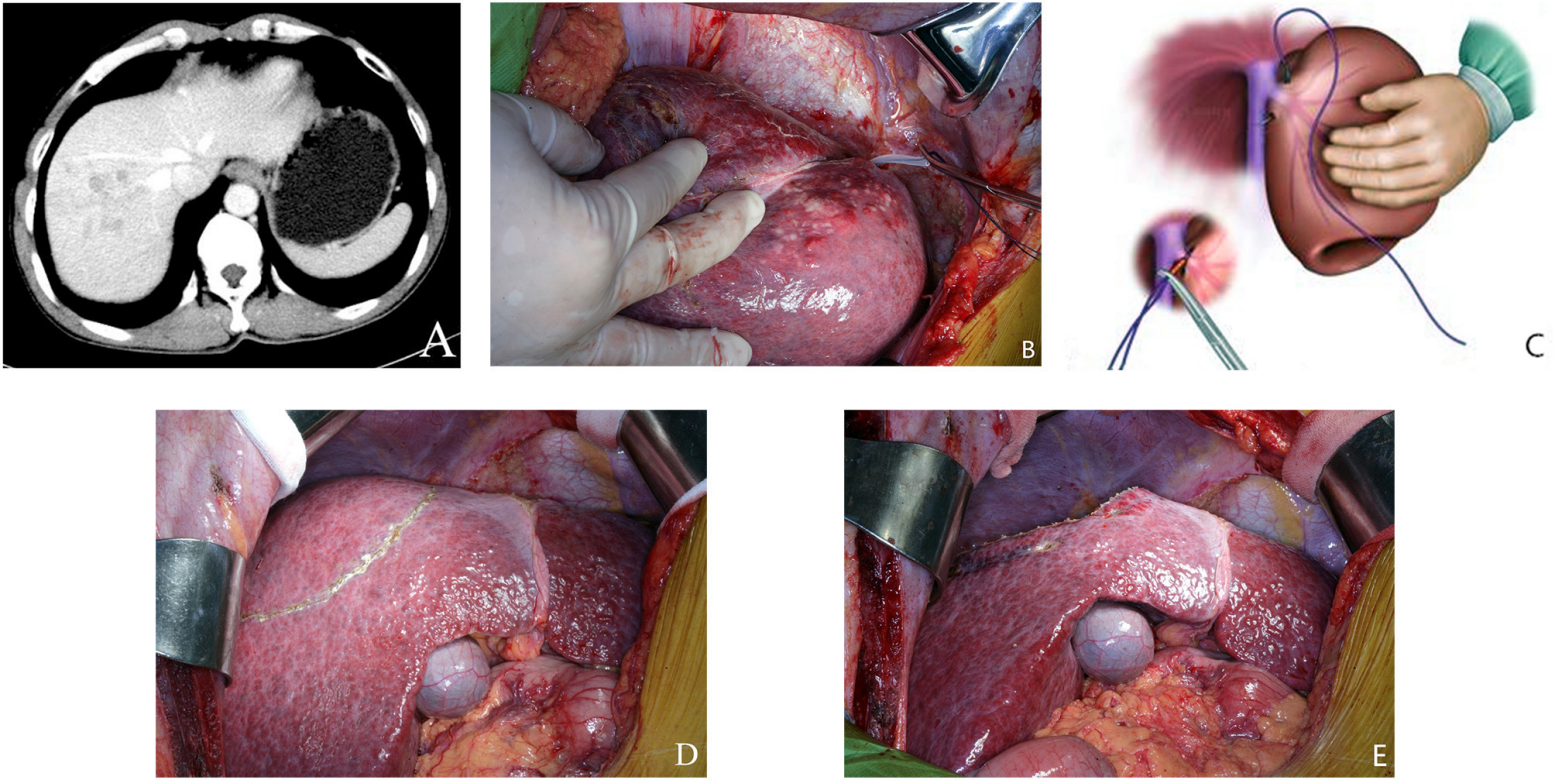
**(A)** Preoperative CT scan of liver. The tumor was located within segment 7–8. **(B)** A new technique using intraoperative ultrasound to guide a blunt needle and a suture to ligate the right hepatic vein. **(C)** A schematic diagram on this maneuver. **(D)** The extent of hepatic resection was shown in the figure. A transection line is positioned on the right side of the middle hepatic vein (MHV) and along the segment 5 branch to preserve them. **(E)** Raw liver surface at the end of bisegmentectomy 7–8.

The study group includes 36 consecutive HCC patients with cirrhosis who underwent bisegmentectomy 7–8 from May 2012 to December 2018, at the Institute of Hepatobiliary Surgery, Southwest Hospital, a tertiary referral university hospital. Each patient in the study group was as closely matched as possible in a 1:1 ratio for a patient who underwent right hemi-hepatectomy in terms of tumor size (±1 cm in diameter), tumor location, number of tumor, age, TNM stage and Child-Pugh classification from the data bank of 1,526 patients who underwent right hemi-hepatectomy for HCC during the study period to form the control group. The diagnosis of HCC and cirrhosis were confirmed by histopathological examination of the operative specimens. The clinicopathological data was retrieved from a prospectively collected computer data bank. The tumor location in both study and control group were all in segments 7–8, with infiltration of right hepatic vein (Figure 1A). There was no tumor nodule elsewhere in the liver. Patients in both groups have not received any other treatments include adjuvant or neoadjuvant chemotherapy, targeted therapy, immunotherapy and so on before operation. And all patients with right hepatic vein invasion by HCC with partial or complete venous blockage were chosen into this study in both groups. The study was approved by the Ethics Committee of the Ethics Committee of the First Affiliated Hospital of Third Military Medical University, PLA. The committee waived the need for individual consent because of the retrospective nature of the study.

All patients followed the same preoperative evaluation protocol which included abdominal ultrasonography, CT scan of the chest and abdomen, CT angiography of hepatic artery, hepatic vein and portal vein, ECG and blood biochemistry. Only 9 patients in the bisegmentectomy group had presence of a prominent inferior RHV shown in contrast-enhanced spiral computed tomography (CT). CT Volumetric studies of liver were routinely performed before surgery. Liver function tests were assessed by the Pugh-Child grading and the indocyanine green clearance test at 15 min (ICG-R15).

### Surgical Planning

Liver CT volumetric studies were routinely done to estimate the future remnant liver volume (FRLV). The ratio of the future liver remnant volume (FLRV%) was calculated by dividing the FLRV by the total liver volume (TLV). The TLV was also calculated by the formula of Urata based on the BSA (TLV^BSA^ = −794.41 + 1267.28 × BSA) (15). It was expressed as a percentage (FLRV% = FLRV × 100/TLV) (15). FRLV% should be more than 50% and also ICG-R15 was ≤14% for right hemi-hepatectomy in cirrhotic patients (9, 16–19). Bisegmentectomy 7–8 also required FRLV% to be more than 50%, and ICG-R15 to be ≤14%. If the patients undergo hemi-hepatectomy, FRLV% will be <40% in study group, bisegmentectomy 7–8 becomes the only viable option. As only liver segment 7–8 were resected, then segment 5–6 were included into the FLR, indications for liver resection in cirrhotic HCC patients were expanded.

Preoperative surgical planning for bisegmentectomy 7–8 also focused on blood inflow and outflow of segments 5 and 6. The right posterior pedicle must be free of tumor involvement and preserved during liver resection. The outflow of segment 6 is the main concern for bisegmentectomy 7–8. A prominent inferior RHV should be confirmed by pre-operative CT scan, or by intraoperative ultrasound, if possible. When no prominent inferior RHV could be detected, a new procedure designed by our team would be carried out to evaluate the venous drainage of the right liver, especially for liver segments 5–6. The right liver was mobilized. The intervenous fossa between the right and the middle hepatic veins was then dissected to give a more accurate point of entry/exit of the needle. Under intraoperative ultrasound guidance, a blunt needle followed by a suture (NO.1, 65 mm, 3/8c, BP-5, ETHICON^®^ Coated VICRYL^®^, Johnson & Johnson Inc.) was used to pass around the right hepatic vein at its confluence into the inferior vena cava through the liver parenchyma. The two ends of the coated Vicryl suture were brought through a short rubber tube, and the rubber tube was pushed against the liver parenchyma to crush the fragile overlying liver tissues to occlude the right hepatic vein (Figures 1B,C). Then venous congestion of segment 5 and segment 6 will be observed for about 5 min. As only selected patients with right hepatic vein invasion by HCC with partial or complete venous blockage were chosen into this study. Mild venous congestion usually occurred in liver segment 6 with obvious color changes on the surface of the right liver. The venous congestion of segment 6 would gradually resolve within 5 min. It is due to short hepatic veins along right border of inferior vena cava had not been dissected or ligated, and segment 5 branch of middle hepatic vein had been preserved.

### Partial Hepatectomy

The operation began with a right subcostal or a midline incision with a right horizontal extension. Routine mobilization of right liver was done by dividing the falciform, right triangular, and coronary ligaments. A self-retaining retractor was then applied. Intraoperative ultrasonography (IOUS) was performed routinely to delineate extent of tumor, rule out tumor nodules in the remnant hepatic parenchyma, determine relationship of tumor with major blood vessels, and mark the plane of hepatic transection (9, 16–19). The first transection line was marked on the liver surface on the right border of the middle hepatic vein (MHV). This line was followed to the origin of segment 8 pedicle which branched from the right anterior sectional pedicle as identified by IOUS. At this point, the transection line was marked by extending it horizontally toward the right side of the liver after identifying the plane between liver segments 6 and 7. This plane was determined by IOUS to identify the origins of the segment 6 and 7 pedicles which branched from the posterior sectional pedicle (Figure 1D). The hepatic pedicles to segment 8 and 7 were isolated and ligated. The right hepatic vein was isolated, ligated and sutured using the technique as previously described. Under intermittent Pringle's maneuver (with cycles of clamping and unclamping of 15/5 min), liver parenchymal transection was carried out using the clamp crushing technique (Figure 1E). During liver parenchymal transection, the central venous blood pressure was maintained at <5 cm H_2_O. Right hemi-hepatectomy was performed following the conventional procedure. The resection margins in both groups were more than 1 cm.

### Statistical Analysis

The end point was the incidence of major postoperative complications and postoperative recurrence. The postoperative recurrence defined as the presence of clinically visible or imaging assessable lesions. RFS is defined as the time from surgery to recurrence. The chi-square test and the Fisher exact test were used to analyze categorical data. The Mann-Whitney U test was used to analyze continuous data with non-normal distributions. The survival curves of the two groups, including the cumulative overall survival and the disease-free survival, were generated by the Kaplan-Meier method. The log-rank test was performed to assess the significance of differences between two groups. All *p*-values reported are two-sided, and *p* < 0.05 was considered statistically significant. Statistical analyses were performed with SPSS23.0 for the Windows computer software (SPSS Inc., Chicago, IL).

## Results

The patient demographics and tumor characteristics are shown in Table 1. There were no significant differences between the two groups of patients. The surgical variables and outcomes, and the postoperative liver function tests are shown in Table 2. There were no significant differences in intraoperative blood loss, blood transfusion, and liver function between the two groups. The median Pringle's maneuver time was similar and was about 1 h in the two groups.

**Table 1:** Patient demographics and tumor characteristics.

**Variable**	**Bisegmentectomy 7–8**	**Right hepatectomy**	***P***^**#**^
No. of patients	36	36	–
Male	25	26	0.756
Age (yrs)^*^	43 (18–75)	45 (20–71)	0.445
Hemoglobin (g/L)^*^	11.5 (8.2–15.5)	12.2 (8.5–16.3)	0.254
Platelet count (10^9^/L)^*^	173 (41–283)	165 (39–311)	0.318
Serum albumin (g/L)^*^	41 (32–51)	42 (28–50)	0.532
Serum total bilirubin (μmol/L)^*^	17 (8–38)	18 (5–39)	0.254
ALT (IU/L)^*^	43 (11–213)	39 (9–233)	0.226
AST (IU/L)^*^	37 (13–198)	35 (11–211)	0.201
Prothrombin time (sec)^*^	13.5 (10.8–17.2)	14.5 (9.5–16.4)	0.189
ICG retention at 15 min (%)^*^	11.3 (3.7–14.8)	8.6 (2.5–10.0)	0.059
HBV carrier	30	31	0.551
Etiology of cirrhosis (HBV/HCV)	30/6	31/5	0.743
Child-Pugh classification			1.000
A	32	31	
B	4	5	
MELD score	16.55 (12–30.36)	16.43 (10.2–40.1)	0.757
Number of tumor			1.000
One	29	29	
Two or more	7	7	
TNM classification			
IIIB	36	36	1.000
Tumor size (cm)	7.5 (4.3–13.4)	7.8 (4.5–14.3)	1.000
Microvascular invasion	10	15	0.200
Satellite nodules	8	10	0.578

* *Value expressed in median with range in parentheses*.

# *Study group compared with control group*.

**Table 2:** Intraoperative parameters and postoperative courses of liver function.

**Clinical parameters**	**Bisegmentectomy 7–8**	**Right hepatectomy**	***P***^***#***^
Intraoperative blood loss (mL)^*^	320 (200–1,200)	350 (200–1,500)	0.235
Intraoperative blood transfusion (mL)^*^	200 (0–800)	300 (0–1,000)	0.457
No. of patients without transfusion (%)	28 (87.5%)	29 (90.6%)	0.688
Operation time (min)^*^	195 (98–305)	201 (119–325)	0.121
Time for Pringle maneuver (min)^*^	59 (35–95)	50 (28–87)	0.101
Serum AST (IU/L)^*^			
On day 3	265 (86–1,268)	248 (66–763)	0.098
On day 7	38 (22–305)	35 (29–266)	0.314
Serum total bilirubin on day 3 (μmol/L)^*^	38 (19–58)	33 (17–57)	0.214
Prothrombin time on day 3 (sec)^*^	15.6 (11.8–18.8)	13.3 (11.1–17.6)	0.287

* *Value expressed in median with range in parentheses*.

# *Bisegmentectomy 7–8 group compared with Right Hepatectomy group*.

The main surgical complications which included hemorrhage, bile leakage, ascites, pleural effusion and liver failure were similar between the two groups (Table 3). The definition of postoperative hemorrhage was that patient has clinical manifestations of shock, furthermore hemoglobin and hematocrit decreased continuously and did not increase after transfusion. The definition of bile leakage was bile continues to flow out or leak in an abnormal way for a longer period of time (≥1 week) after surgery, and the level of bilirubin in postoperative abdominal drainage fluid is more than 3 times that of blood bilirubin in the same period. Ascites and pleural effusion all confirm by ultrasound or CT. Liver failure was defined as a postoperatively acquired functional disorder in the ability of the liver to maintain its synthetic, excretory, and detoxifying functions, which are characterized by an increased INR together with hyperbilirubinemia on or after postoperative day 5 (20). Hospital mortality means the rate of death from any cause in hospitalized populations. There were no significant differences in the overall morbidity rates (*P* = 0.756), and in the morbidity rates classified according to Clavien's classification (*P* = 0.776). And after puncture drain, patients with hydrothorax and bile leakage get well soon. There was no in-hospital or postoperative 90 day mortality in this study.

**Table 3:** Postoperative complications and hospital stay.

**Clinical parameters**	**Bisegmentectomy 7–8**	**Right hepatectomy**	***P***^***#***^
No. of major complications	6	7	0.756
Postsurgical hemorrhage	1	1	
Bile leakage	2	2	
Ascites	1	2	
Pleural effusion	2	2	
Liver failure	0	0	
Hospital mortality	0	0	
Clavien's classification of surgical complications	6 (18.8%)	9 (28.1%)	0.776
Grade I	4	5	
Grade II	1	2	
Grade III	1	1	
Grade IV	0	1	
Grade V	0	0	
Postoperative hospital stay (days)	10 (8-21)	11 (8-32)	0.224

# *Bisegmentectomy 7–8 group compared with Right Hepatectomy group*.

**Figure 2:**
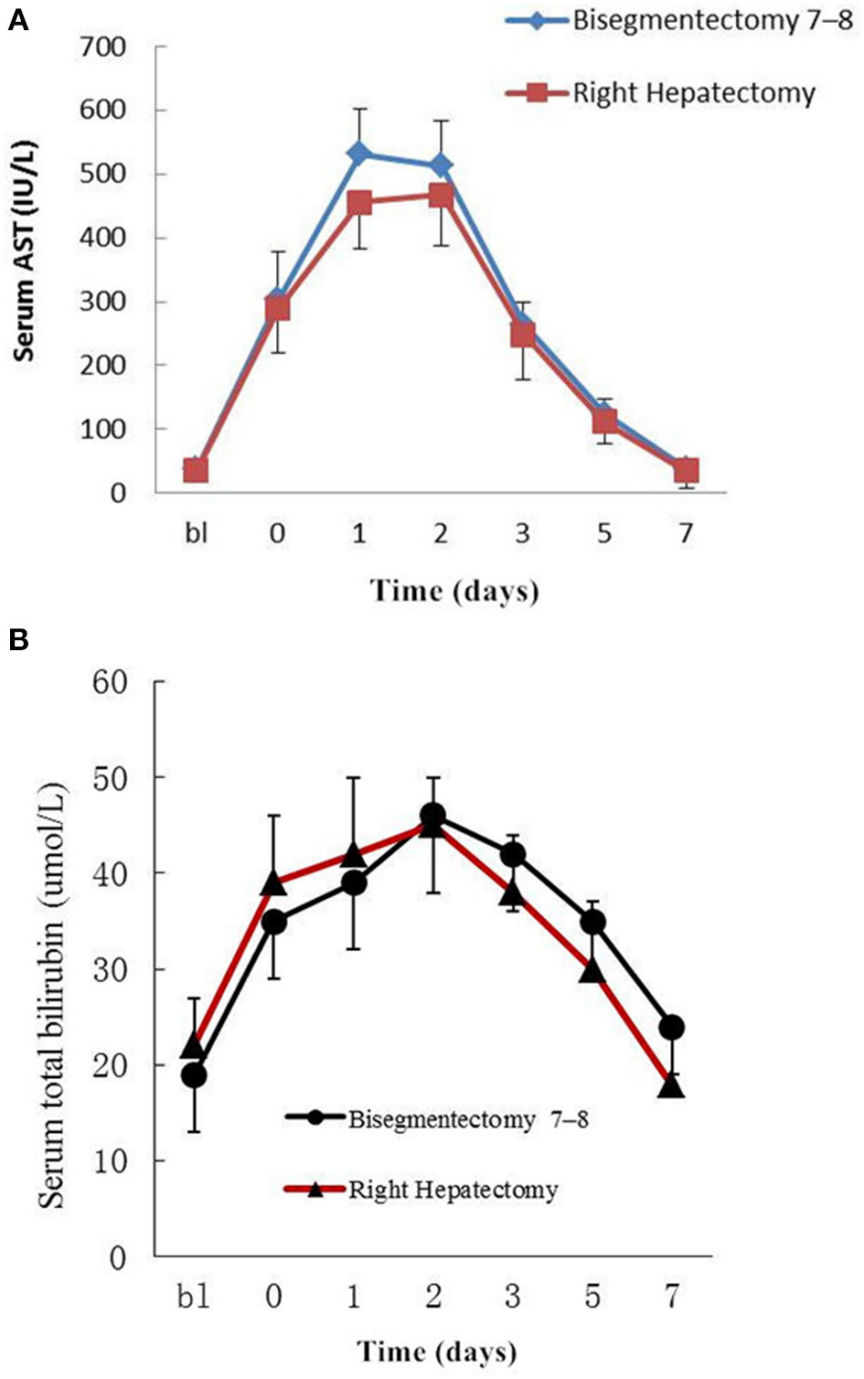
**(A)** Peri-operative changes of serum AST. bl: Preoperative value as baseline; 0–7: Postoperative days; Data are mean ± SEM. *P* = 0.054. **(B)** Peri-operative changes of serum total bilirubin. bl: Preoperative value as baseline; 0–7:Postoperative days; Data are mean ± SEM. *P* = 0.610.

The changes in the perioperative serum AST and total bilirubin are shown in Figure 2. The serum AST rose rapidly to a peak on the first and second days after bisegmentectomy 7–8 and right hepatectomy, respectively, then decreased gradually to normal (Figure 2A). There was no significant difference in the perioperative average serum AST between the two groups (*P* = 0.054). Although the postoperative serum AST on postoperative day 1 in the study group was higher than the control group, the difference was not significant (*P* = 0.087). The perioperative serum total bilirubin also rose gradually after surgery, then dropped to normal (Figure 2B). There was no significant difference in the perioperative average total bilirubin levels between the two groups (*P* = 0.610).

**Figure 3:**
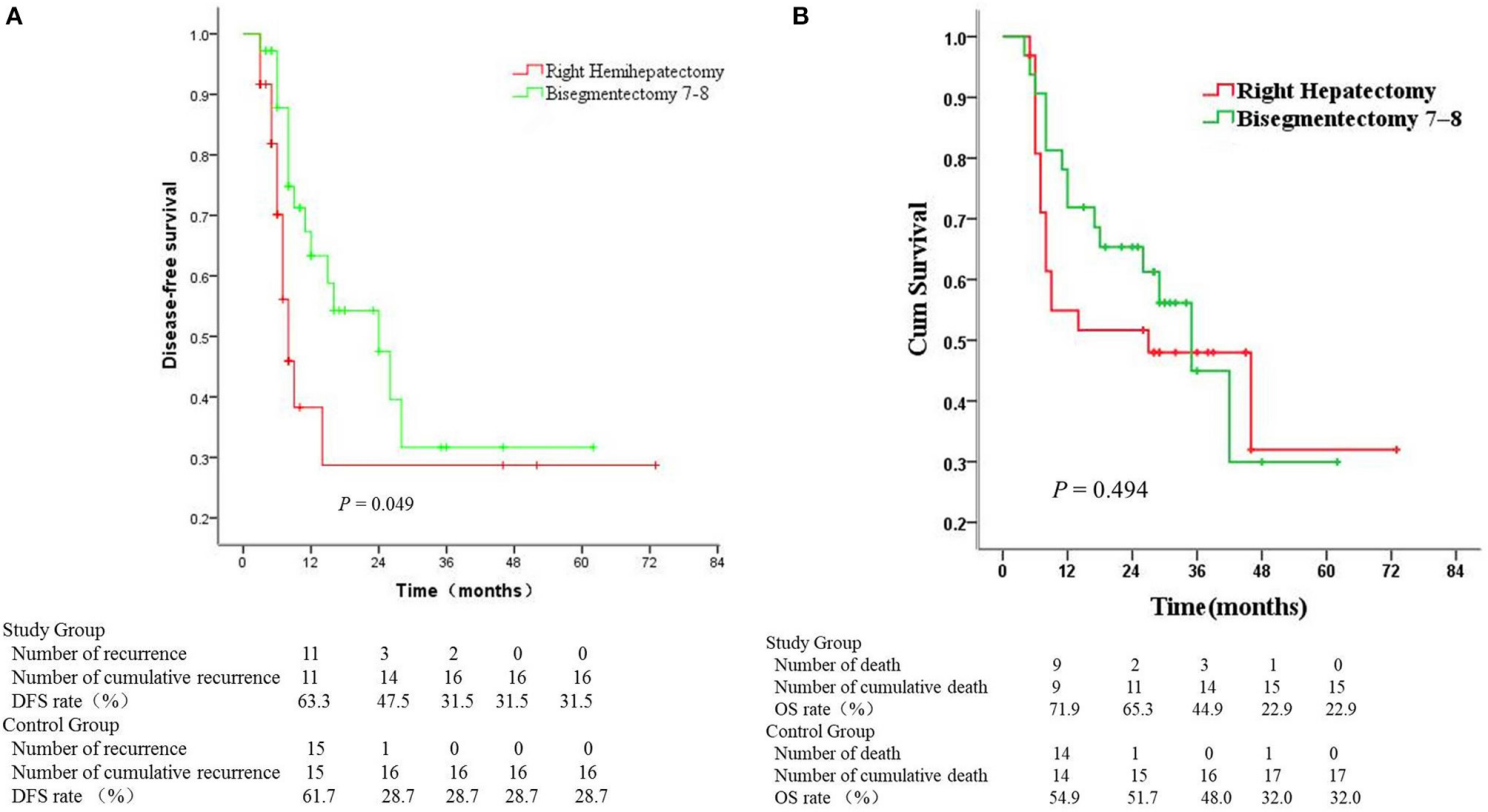
Survival analysis in the Bisegmentectomy 7–8 Group and Right Hepatectomy Group. **(A)** Disease-free survival; **(B)** Cumulative overall survival (Bisegmentectomy 7–8 vs. Right Hepatectomy; Kaplan–Meier, log–rank test).

The mean follow-up was 30 and 32 months for the study and control group, respectively. The DFS rates in the study group were just significantly better than the control group (*P* = 0.049) (Figure 3A). The median DFS was 24 months for the study group and 8 months for the control group. Meanwhile, the median cumulative overall survival was 35 months for the study group and 27 months for the control group (*P* = 0.494). There was no significant difference in the cumulative overall survival between the two groups (Figure 3B). For patients with intrahepatic recurrence, we performed repeat resection or radiofrequency ablation if possible. TACE was given when multiple recurrent intrahepatic lesions were confined.

## Discussion

HCC is one of the most common malignant tumors in the world (2). At present, HCC ranks as the second cause of cancer death in China. Most cases of HCC are closely associated with chronic hepatitis B, especially post-hepatitis B cirrhosis (21, 22). Although molecular targeted therapy and immunotherapy for HCC have developed rapidly, surgery, either in the form of hepatic resection or liver transplantation, remains the most effective curative treatment (8, 22–24).

Tumors localized in segments 7–8 in patients without a prominent right inferior right hepatic vein shown on contrast enhanced CT scans are usually treated with right hemi-hepatectomy, as after bisegmentectomy 7–8, the remnant segments 5 and 6 may lack venous drainage. Nakamura and Tsuzuki (25) advocated bisegmentectomy 7–8 only when there is a large RHV with small accessories veins. Others have shown that bisegmentectomy 7–8 is feasible and safe when there is a large right inferior hepatic vein (26–28). However, when a large right inferior hepatic vein is absent, controversies exist on the safety and feasibility of bisegmentectomy 7–8 (26, 29). In our study, only selected patients with right hepatic vein invasion by HCC with partial or complete venous blockage were chosen and this selection criterion is firstly reported. And only 9 patients in the bisegmentectomy group with presence of a prominent inferior RHV shown on CT, but none of our 36 patients developed postoperative liver failure (POLF). The results of our study are consistent with the observations of some previously published reports (29–31). Thus, the existence of a large inferior right hepatic vein may not be the only factor in preventing venous outflow stasis in segments 5 and 6 after bisegmentectomy 7–8. In this study, patients with right hepatic vein invasion by tumor were chosen so that the venous drainage of segments 5, 6, into right hepatic vein had already been partially or completely occluded before operation. The preservation of short hepatic veins along right border of inferior vena cava, and careful preservation of terminal branches of middle hepatic vein resulted in reestablishment of adequate venous drainage of segments 5, 6 after bisegmentectomy 7–8. And another reason is the presence of communicating veins between adjacent hepatic veins (32, 33).

A new occlusion technique was performed in this study to ligate the right hepatic vein at its confluence with inferior vena cava before performing liver parenchymal transection. This was also used to judge the venous drainage of the right liver before hepatic parenchyma transection, and to reduce blood loss during hepatic parenchymal transection. Because of the application of this new method, it avoids the complex operation of dissecting the hepatic vein, reduces the probability of intraoperative bleeding and the operation time. Especially when liver cancer patients with severe cirrhosis undergoing hepatectomy, the longer the operation time, the more bleeding, the greater the probability of serious complications such as liver failure. The application of this new method can undoubtedly benefit patients. The results of this study suggested that bisegmentectomy 7–8 to be safe and feasible, and did not increase the risk of surgery and the incidence of postoperative complications in carefully selected patients.

Bisegmentectomy 7–8 which preserved more liver parenchyma may increases resectability in cirrhotic patients with HCC, increases the chance of “curative” therapy by repeat hepatectomy in future tumor recurrence. Especially for patients who perform right hemi-hepatectomy and reserve liver volume is not enough, bisegmentectomy 7–8 can improve their prognosis. Maybe the resection margins were not larger than right hemi-hepatectomy. However, some studies have shown that increasing the margins of resection does not improve the possibility of recurrence of hepatocellular carcinoma, and biologic tumor characteristics are the principal factors predictive of local and systemic recurrence (34). The results of long-term survival outcomes in this study showed that there was no significant difference in the cumulative overall survival between the two groups. And the median cumulative overall survival in study group was longer than the control group (35 vs. 27 mo). The absence of a prominent inferior right hepatic vein should not be an absolute contraindication for bisegmentectomy 7–8. Bisegmentectomy 7–8 should be performed more often than is now reported for selected cirrhosis patients. The recurrence and mortality rate of these patients were higher than that of the common patients, as a result of the patients in the study and control group were all HCC with cirrhosis. And all patients in both groups existed right hepatic vein invasion by HCC. The numbers of patients with MVI were 10 and 15, respectively. These are risk factors for postoperative recurrence (35–37). Furthermore, the first and second years were the periods of high recurrence, due to biological characteristics of HCC.

The limitations of this study are: First, this is a small sample retrospective study on prospectively collected data. There are intrinsic defects of such a type of study. Second, this study is mainly on HCC with a background of chronic hepatitis B cirrhosis. Whether the results of this study can be extrapolated to HCC of other etiologies are unknown. Third, surgeons involved in this study are liver surgeons who are experienced in cirrhotic liver resection. The results of this study might not be generalized to less experienced surgeons.

In conclusion, bisegmentectomy 7–8 was safe and feasible for selected cirrhosis patients, and did not increase the perioperative risk and inferior long-term overall survival outcomes. It extended the indications for liver resection in patients with borderline volumes of future liver remnant for cirrhotic HCC patients.

## Data Availability Statement

The original contributions presented in the study are included in the article/supplementary material, further inquiries can be directed to the corresponding author/s.

## Ethics Statement

The studies involving human participants were reviewed and approved by the Ethics Committee of the First Affiliated Hospital of Third Military Medical University, PLA. Written informed consent for participation was not required for this study in accordance with the national legislation and the institutional requirements. Written informed consent was obtained from the individual(s) for the publication of any potentially identifiable images or data included in this article.

## Author Contributions

XW and HH: conceptualization, formal analysis, investigation, writing—original draft, writing—review and editing, and visualization. YL: formal analysis, investigation, and visualization. SC: investigation and formal analysis. KM and KF: investigation and writing—review and editing. WL: conceptualization, investigation, data curation, writing—review and editing, supervision, and project administration. FX: conceptualization, investigation, data curation, writing—review and editing, supervision, funding acquisition, and project administration. All authors contributed to the article and approved the submitted version.

## Conflict of Interest

The authors declare that the research was conducted in the absence of any commercial or financial relationships that could be construed as a potential conflict of interest.
